# Evidence Supporting the Involvement of the Minority Compounds of Extra Virgin Olive Oil, through Gut Microbiota Modulation, in Some of the Dietary Benefits Related to Metabolic Syndrome in Comparison to Butter

**DOI:** 10.3390/molecules28052265

**Published:** 2023-02-28

**Authors:** María Collado Olid, Marina Hidalgo, Isabel Prieto, Antonio Cobo, Ana M. Martínez-Rodríguez, Ana Belén Segarra, Manuel Ramírez-Sánchez, Antonio Gálvez, Magdalena Martínez-Cañamero

**Affiliations:** 1 Área de Microbiología, Departamento de Ciencias de la Salud, Universidad de Jaén, 23071 Jaén, Spain; 2 Área de Fisiología, Departamento de Ciencias de la Salud, Universidad de Jaén, 23071 Jaén, Spain; 3 Departamento de Estadística e Investigación Operativa, Universidad de Jaén, 23071 Jaén, Spain

**Keywords:** virgin olive oil, refined olive oil, butter, gut microbiota, systolic blood pressure, next generation sequencing

## Abstract

Extra virgin olive oil (EVOO) has proven to yield a better health outcome than other saturated fats widely used in the Western diet, including a distinct dysbiosis-preventive modulation of gut microbiota. Besides its high content in unsaturated fatty acids, EVOO also has an unsaponifiable polyphenol-enriched fraction that is lost when undergoing a depurative process that gives place to refined olive oil (ROO). Comparing the effects of both oils on the intestinal microbiota of mice can help us determine which benefits of EVOO are due to the unsaturated fatty acids, which remain the same in both, and which benefits are a consequence of its minority compounds, mainly polyphenols. In this work, we study these variations after only six weeks of diet, when physiological changes are not appreciated yet but intestinal microbial alterations can already be detected. Some of these bacterial deviations correlate in multiple regression models with ulterior physiological values, at twelve weeks of diet, including systolic blood pressure. Comparison between the EVOO and ROO diets reveals that some of these correlations can be explained by the type of fat that is present in the diet, while in other cases, such as the genus *Desulfovibrio*, can be better understood if the antimicrobial role of the virgin olive oil polyphenols is considered.

## 1. Introduction

For several years, studies have dealt with the influence of the intestinal microbiota and the health of the gut environment on the development of obesity, metabolic, and even neurodegenerative diseases through the gut–brain axis [1,2,3]. Diet is one of the main drivers in the microbiota composition and it is known that high-fat diets have a negative effect on health by exerting an action on the microbial taxa in the intestine which are essential for host homeostasis maintenance [4]. Changes in the gut microbiota, microbial compound production, and mucosal immune system function can contribute to cardiovascular, metabolic, and cognitive health [5].

Olive oil is not the most widely consumed oil in the world (palm and soybean oils lead to the global consumption of vegetable oils), but it has always been the principal fat added to the Mediterranean diet. Extra virgin olive oil (EVOO) is a functional food with a wide variety of healthy components, such as monounsaturated fatty acids and phenolic compounds, with beneficial effects on cardiovascular diseases due to their anti-inflammatory and antioxidant activities [6], and it has been shown that this effect is at least in part exerted along with modulation of the gut microbiota [7,8,9].

Several studies propose that the anti-inflammatory, antitumor, antioxidant, and modulatory effects of the intestinal microbiota (associated with this diet and the EVOO) on chronic inflammatory diseases could be attributed to polyphenols [revised in [10,11]]. In order to address this issue, it is interesting to compare oils with different levels of polyphenols. This can be easily undertaken because only virgin olive oils have intact polyphenol content, while other commercial olive oils, the refined ones, have lost them. EVOO is mechanically extracted directly from olives without chemical intervention, and it contains relatively high amounts of phenolic compounds and tocopherols. To improve its palatability, low-quality virgin oil undergoes chemical treatment, transforming it into refined olive oil (ROO), which has the same composition of fatty acids as EVOO but loses the minor compounds [12,13,14,15].

There are few studies about the influence of ROO on health and none on the intestinal microbiota. Because of this, our research team set out to study this fact, finding different behavior in ROO compared to EVOO as well as a different microbial profile when analyzed by denaturing gradient gel electrophoresis (DGGE) [5]. Subsequently, a metagenomic study of 16S ribosomal DNA showed clear differences in both fecal microbiota and physiological variables related to metabolic syndrome when we compared different high-fat diets (HFDs) enriched with butter, ROO, and EVOO versus the standard diet. For this, mice were fed standard or enriched chow with the different HFDs for twelve weeks, and physiological variables and fecal microbial content were measured at the end of the experiment [16]. We clearly showed a significant statistical relationship by using multiple regression models between a specific diet, physiological variables, and different taxa, and the comparison between the effects of the two olive oils allowed us to propose which EVOO-involved correlations were driven by the polyphenol content and which ones were not.

However, correlation is not causation, and in order to clarify if variations in the microbial taxa had an effect on their correlating physiological variables or if they both just changed concomitantly under the impact of the diet, we studied the results obtained in the middle of the experiment, after only six weeks of diets, comparing them with each other and also with the ones obtained at twelve weeks. Results comparing EVOO and butter at six weeks showed that several changes in the microbial composition at this time point have regression coefficients that are statistically significant with metabolic and physiological variables at twelve weeks, even though some of the taxa did not maintain statistical significance by the end of the experiment [17]. This is important because these changes can be used as markers of future risks.

In the current research, the results obtained at six weeks under the EVOO and the ROO diets were compared in order to find out which of these EVOO-driven differences have their origin in the polyphenol content lost in ROO, and the data could give information on the possible role of the intestinal microbiota in the final outcome of these diets.

## 2. Results

### 2.1. Physiological Parameters

**Figure 1:**
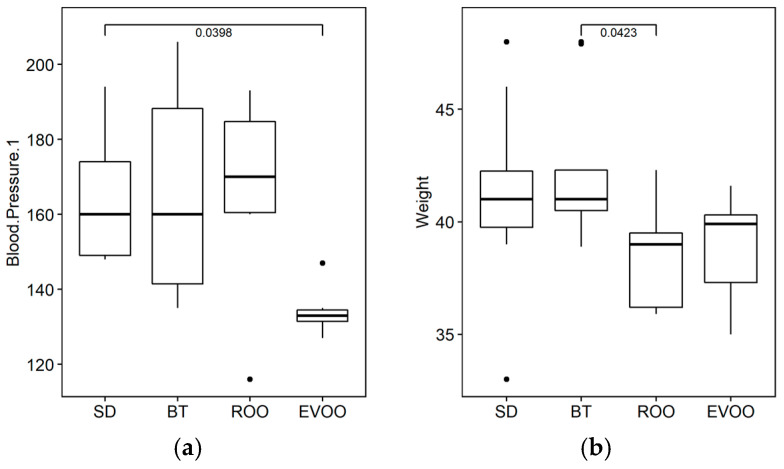
Physiological means found to be statistically different with a signification at 5% ((**a**), blood pressure, *p* = 0.004) and 10% ((**b**), weight, *p* = 0.057) after six weeks of diet. BT, butter enriched diet; EVOO, extra virgin olive oil enriched diet; ROO, refined olive oil diet; SD, standard diet.

Food intake, water intake, diuresis, body weight, and systolic blood pressure were measured after six weeks of the experimental period. The only significant difference was found in the systolic blood pressure, where the EVOO diet showed significantly lower values than the other diets, including the ROO diet, after applying a robust ANOVA test using R (one-way analysis of means and not assuming equal variances), with a *p*-value of 0.004 (Figure 1a). Weight did not show significant differences at 5%, but it did at 10% (*p* = 0.057), with lower values in EVOO and also in ROO (ANOVA test) (Figure 1b), with an average dietary intake of 3.8 g/day and weight gain of 7.9 g.

### 2.2. Sequencing, Taxa Adscription, Percentage Comparison and Multiple Regression Models

After sequencing the 35 samples until the number of reads was stable, and after trimming and filtering, 353,260 sequences were obtained with a mean length between 548 and 575 nt and a total amount of 196.91 MB. In order to associate an organism with each sequence obtained, a blast search was performed, and the reads were grouped based on family, genera, and species levels. In total, sequences were classified into 9 phyla, 89 families, 227 genera, and 538 species.

**Figure 2:**
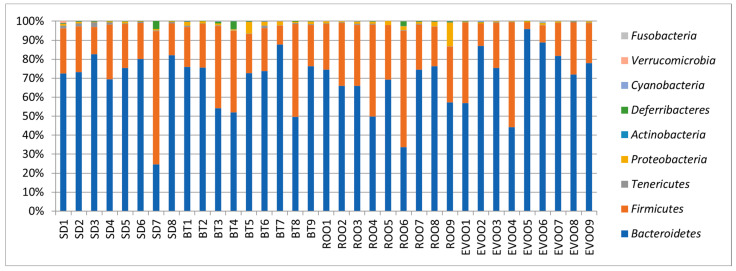
Global bacterial phyla distribution for each diet as a percentage of total sequences retrieved from fecal samples after six weeks of diet. Each column corresponds to one animal fed a diet enriched with butter (BT), extra virgin olive oil (EVOO), refined olive oil (ROO), or standard chow (SD). SD, EVOO, and BT diet values from [17].

**Figure 3:**
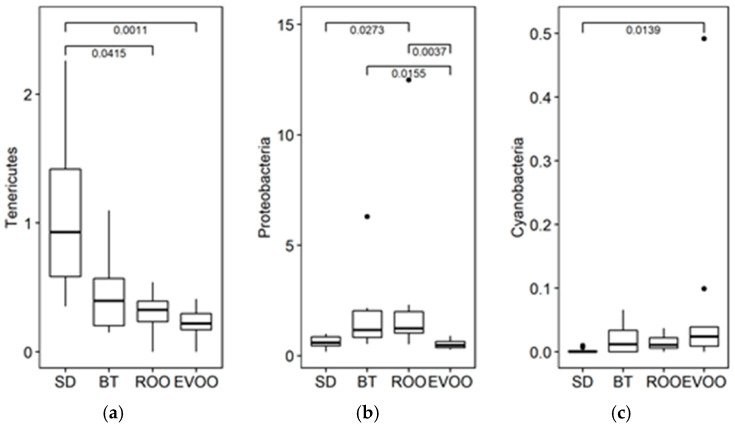
Box plot representation of the percentage distribution of the phyla (**a**) *Tenericutes*, (**b**) *Proteobacteria*, (**c**) *Cyanobacteria*, with statistically significant differences in fecal samples from mice fed a diet enriched with butter (BT), extra virgin olive oil (EVOO), refined olive oil (ROO), or standard chow (SD) for six weeks. SD, EVOO, and BT diet values from [17].

When a Kruskal–Wallis test was used to check if the distributions of the diverse phyla (Figure 2) were the same among the four diets, the phyla *Tenericutes* and *Proteobacteria* showed significant differences (*p* = 0.002 and *p* = 0.0005, respectively, Figure 3). Multiple pairwise comparisons were performed showing that there are statistically significant differences, being EVOO and ROO mean values lower than those of SD in the first case, while in the case of *Proteobacteria*, EVOO values were lower than both BT and ROO, ROO also being higher than SD. *Cyanobacteria* also showed differences (*p* = 0.02) with greater values in EVOO against SD (Figure 3). However, the high number of data points with a value of 0 in this phylum makes its significance more unreliable. A multiple linear regression analysis was performed for each physiological variable considering the phyla as independent variables, and we only obtained a model for systolic blood pressure (R^2^ = 0.65; *p* = 0.0076) with the phyla *Tenericutes* and *Proteobacteria* (regression coefficient estimates of −227.30 and −247.63; s.e. of ±72.24 and ±72.82; and *p*-values of 0.0059 and 0.0034, respectively). Since we also had available the physiological measurements at the end of the experiment, already reported in Prieto et al. [9] and Martínez et al. [16], we repeated the regression analysis with these variables as dependent ones, but no statistically significant results were obtained.

**Figure 4:**
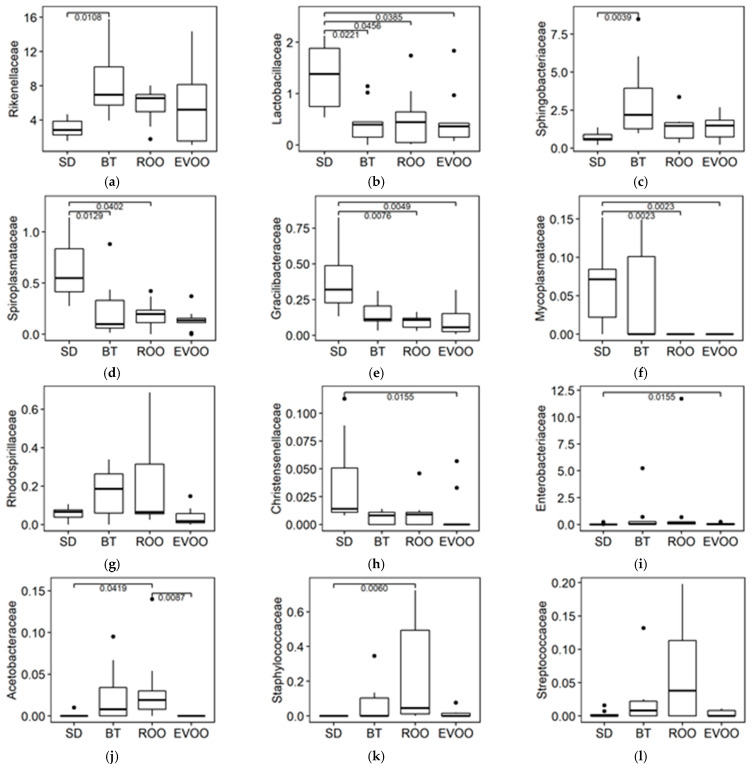
Box plot representation of the percentage distribution of the twelve families (**a**) *Rikenellaceae*, (**b**) *Lactobacillaceae*, (**c**) *Sphingobacteriaceae*, (**d**) *Spiroplasmataceae*, (**e**) *Gracillibacteraceae*, (**f**) *Mycoplasmatace*, (**g**) *Rhodospirillaceae*, (**h**) *Christensenellaceae*, (**i**) *Enterobacteriaceae*, (**j**) *Acetobacteraceae*, (**k**) *Staphylococcaceae*, (**l**) *Streptococcaceae*, with significant differences (*p* < 0.05) among fecal samples from mice fed a diet enriched with butter (BT), extra virgin olive oil (EVOO), refined olive oil (ROO), or standard chow (SD). Adjusted *p*-values of significant pairwise comparisons are also shown. SD, EVOO, and BT diet values from [17].

The same procedure was repeated at the family level. According to the Kruskal–Wallis test, out of the 89 families that were detected, twelve of them showed statistically significant differences among the four diets; in eight of them, the ROO diet was significantly different in a multiple pairwise analysis, but only in one case (fam. *Acetobacteriaceae*) was it significantly different from the EVOO diet. Figure 4 displays the box plots of these twelve families with multiple pairwise comparisons. Different multiple linear regression models were fitted to explain each physiological variable (both at 6 and 12 weeks) using as independent variables all the families with significant differences. Ten of them were involved in models with data on physiological variables from the two time points (Table 1).

**Figure 5:**
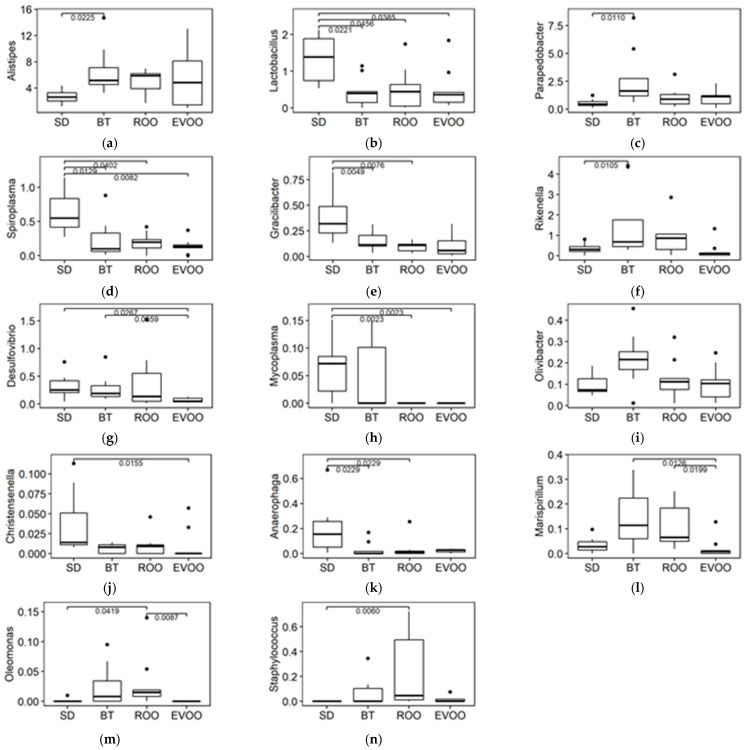
Box plot representation of the percentage distribution of the fourteen genera (**a**) *Alistipes*, (**b**) *Lactobacillus*, (**c**) *Parapedobacter*, (**d**) *Spiroplasma*, (**e**) *Gracillibacter*, (**f**) *Rikenella*, (**g**) *Desulfovibrio*, (**h**) *Mycoplasma*, (**i**) *Olivibacter*, (**j**) *Christensenella*, (**k**) *Anaerophaga*, (**l**) *Marispirillum*, (**m**) *Oleomonas*, (**n**) *Staphylococcus*, with significant differences (*p* < 0.05) among fecal samples from mice fed a diet enriched with butter (BT), extra virgin olive oil (EVOO), refined olive oil (ROO), or standard chow (SD). Adjusted *p*-values of significant pairwise comparisons are also shown. SD, EVOO, and BT diet values from [17].

We also compared the prevalence of the 227 genera obtained when studying the four diets. In this case, the Kruskal–Wallis test results indicated that fourteen of them had statistically significant differences, which are shown in Figure 5. When applying multiple pairwise comparisons, EVOO and ROO had the same significant differences only in three genera. With respect to the rest of them, in two genera both oils were significantly different from each other; in another two, only EVOO was involved in a significantly different pair; and, finally, in three more genera, ROO was the only one of the two to be statistically significant. Table 2 shows the results found after applying a multiple linear regression analysis using the genera with significant differences.

**Figure 6:**
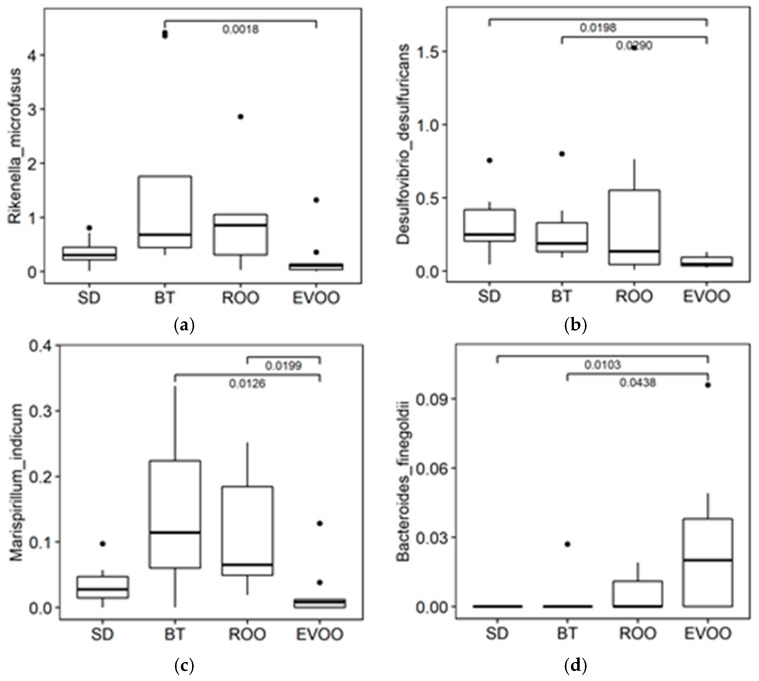
Box plot representation of the percentage distribution of the four species (**a**) *Rikenella microfusus*, (**b**) *Desulfovibrio desulfuricans*, (**c**) *Marispirillum indicum*, (**d**) *Bacteroides finegoldii*, with significant differences (*p* < 0.05) between mice fed a diet enriched with butter (BT) and a diet enriched in virgin olive oil (EVOO). SD, standard chow. ROO, refined olive oil.

Finally, species were also studied. In this case, since the number of species was too high, we centered our attention on those showing differences with statistical significance between BT and EVOO in order to check how ROO clustered in those cases. Four species fulfilled this premise, and the comparisons are shown in Figure 6. ROO and EVOO did not behave in the same way in any of them and in one case (*Marispirillum indicum*), EVOO was statistically significantly different from BT and ROO. Multiple regression models were drawn and only three were found that fully complied with all the hypotheses required, one on SBP at six weeks that involved *Bacteroides finegoldii* (R^2^ = 0.16; *p* = 0.0439; regression coefficient estimates of −2.83; s.e. of ±1.33; and *p*-value of 0.0439) and two at 12 weeks for FI, involving *Rikenella microfusus* (R^2^ = 0.13; *p* = 0.0360; regression coefficient estimates of −0.45; s.e. of ±0.20; and *p*-value of 0.0361) and for WI involving *Marispirillum indicum* (R^2^ = 0.15; *p* = 0.0317; regression coefficient estimates of −2.06; s.e. of ±0.91; and *p*-value of 0.0317).

## 3. Discussion

Previously, we have shown that different high-fat diets have diverse effects on the intestinal microbiota of mice [7]. We have also shown that this effect correlates with physiological variables related to the metabolic syndrome, with systolic blood pressure being the most noticeable, correlating with the percentage of *Desulfovibrio* sequences in feces, increasing under a butter-enriched diet but not doing so under an EVOO-enriched one [9]. Consequently, we compared these outcomes with the physiological and microbiological profiles of mice under a refined olive oil-enriched diet, finding that both olive oils had dissimilar effects on half the families with statistically significant differences [16], including fam. *Desulfovibrionaceae*, being again the genus *Desulfovibrio* preeminent since EVOO and ROO had an opposite influence on its percentage in a multiple pairwise comparison analysis. These data allowed us to discuss whether EVOO polyphenols and unsaponifiable matter, in general, could be responsible for all the EVOO effects on the bacterial taxa that were not duplicated in the ROO experimental group.

All these results were measured after 12 weeks of diet when physiological changes were more visible. However, we had the opportunity to study the microbial intestinal environment and the physiological health of the same mice but six weeks earlier, in the midterm balance of the experiment. This analysis was performed, and comparisons and multiple regression models were drawn between the SD, BT, and EVOO groups not only at six weeks but also with the physiological and metabolic variables measured at the end of the experiment at 12 weeks [17]. Certainly, at six weeks, physiological changes are not appreciated yet, although we can already clearly detect intestinal microbial changes since these are much less subject to the global homeostasis of the organism. Remarkably, these changes in the intestinal microbiota not only had a statistically significant relationship with the concomitant physiological variables, but they also did with variables measured at the end of the experiment. Once more, *Desulfovibrio* had an important role in the discussion because it was again less prominent in the EVOO group at six weeks and showed a significant regression coefficient with body weight, food intake, water intake, and systolic blood pressure measured at 12 weeks of diet.

In the work we present now, we have made the analysis, adding the data obtained in the group of mice fed a refined olive oil-enriched diet at six weeks of experiment in order to derive the role of the polyphenols at this time point in the feeding process. The aim is to uncover which changes in the intestinal microbiota prompted by an EVOO diet at this time are due to its fatty acid composition and which ones are influenced by its minority compounds, and moreover, if these relations have any manifestation after a further six weeks.

With respect to the physiological variables, the only significant difference is found in systolic blood pressure, and in a relative manner. We had already reported significantly low SBP values in the EVOO group with respect to the BT and SD groups, neither of which maintained excessive SBP nor a difference between them [17]. If this effect were to be dependent on the degree of saturation of the fatty acids, it would have been logical to expect that ROO data would be similar to EVOO. However, in our current results, the ROO group clusters with the SBP values of BT and SD, which is significantly different from the EVOO data. Following this variable till the end of the experiment at 12 weeks, the blood pressure values will have evolved to be significantly higher in the BT group with respect to the three other diets (SD, EVOO, and ROO). Probably, then, in the long run, the saturation of the fatty acids may have a greater weight than the polyphenol or minor component effects.

When studying the intestinal microbiota at the phylum level, ROO behaves like EVOO in the *Tenericutes* profile and unlike it in *Proteobacteria*, where ROO clusters with BT. *Proteobacteria* is quite dominant in the butter group at twelve weeks [9]. At the family level, out of the twelve families with significant differences, only in one of them were EVOO and ROO significantly different from each other (*Acetobacteriaceae*), while in the other four, either EVOO (*Christensenellaceae* and *Enterobacteraceae*) or ROO (*Spiroplasmataceae* and *Staphylococcaceae*) was significantly different from SD while the other unsaturated fat was not, even though the two fat profiles were similar. In the rest of the families, seven in total, EVOO and ROO had an identical statistical outcome. Moreover, in four of these seven families (*Rikenellaceae*, *Sphingobactereaceae*, *Gracillibacteraceae,* and *Mycoplasmataceae*), BT behaved statistically different from both EVOO and ROO, while in another (*Lactobacillaceae*), the three high-fat diet groups had percentages significantly lower than the SD group, indicating a probable common effect of all types of fat. Six weeks later, at the end of the experiment, the scenario would be totally different, with further differences between both EVOO and ROO in the predominance of the bacterial taxa [16]. This fact could be indicative of the relatively limited role of the polyphenols in the effects of virgin olive oil on the intestinal microbiota in a short-term diet in contrast to a long-term diet in these cases.

With respect to the regression models found using the significant families as an independent factor, most of the significant regressions already reported in Andújar et al. [17] are maintained, but some are not. Thus, in the 12-week body weight model, only *Sphingobacteriaceae* and *Spiroplasmataceae* are maintained; the model for diuresis keeps only *Rhodospirillaceae* at six weeks, and the model for blood pressure at twelve weeks maintains only the family *Sphingobacteriaceae*, although in the case of fam. *Spiroplasmataceae*, its genus, *Spiroplasma,* also maintains the statistical significance. Among the regression coefficients that maintain their statistical significance, it is worth noting the relation of the families *Lactobacillaceae* (direct) and *Gracillibacteraceae* (inverse) with food intake both at six and at twelve weeks of diet. The fact of losing the significance of some regression coefficients does not diminish their importance in the other three diets because, by including a new diet, new uncontrolled factors can be introduced as well. However, if they are maintained, the result is more robust. In this way, the relationship of lactic acid bacteria with food intake all through the experiment, with and without ROO, makes this a very interesting result, especially because it is not included in the regression model for body weight at any point. However, lactobacilli have previously been related to obesity in humans [18], and this could reflect some mechanism that is no longer adjusted in overfed modern societies. On the contrary, *Gracillibacteraceae* has a direct relationship with body weight at six weeks but a negative one with food intake both at six and 12 weeks, and this result could be related to some kind of regulation when the animal has reached the appropriate weight. In any case, in both families, EVOO and ROO have the same effect, so these outcomes cannot be attributed to the minority compounds. Moreover, in *Lactobacillaceae,* the three high-fat diets have the same statistical behavior, so it could confirm the effect of HFD in general as discussed previously with only two types of fat [17].

This is not the case for fam. *Acetobacteraceae* and its genus *Oleomonas*, whose percentage is significantly lower in the EVOO diet than in the ROO group, with the BT percentage being closer to ROO and the SD percentage being closer to EVOO. The predominance of this family is low, but it is worth mentioning because it is the only one where the two olive oils have a statistically significant opposite profile. This family also happens to correlate with body weight at six weeks and inversely with food intake and water intake at twelve weeks, both without ROO [17] and with ROO (this work). Although very distantly phylogenetically related, fam. *Acetobacteraceae* shares with *Sphingobacteriaceae* the presence in its membrane of a lipid type that is rare in bacteria, sphingolipids [19]. They can be obtained directly from the diet but can also be synthesized from palmitic acid [20]. In this sense, they can be easily found in butter but also as components of the minor polar lipid fraction in olive oil [21], where palmitic acid can also be detected as part of the fatty acid profile [22]. This could explain the higher presence of this family in BT and ROO and their low profile in the standard diet. According to its lipid composition, EVOO would be expected to share ROO results, and the fact of not doing so can be explained by the impact of the virgin olive oil polyphenols and their antimicrobial effect.

When widening our analysis to the genus level, most of the genera with significant differences resembled the characteristics of their families, as discussed above. There are, though, two cases worth reviewing for two different reasons: *Rikenella* and *Desulfovibrio*. *Rikenella* is taxonomically framed in the homonym family, *Rikenellaceae*, but in this study, both taxa do not share the same profile, and it is another same family genus, *Alistipes*, which is much more prominent in percentage and the one that maintains the family outcome (Figure 5). While fam. *Rikenellaceae* and gen. *Alistipes* present high values and variability in EVOO, gen. *Rikenella* has very low values in this diet, presenting significant differences between the EVOO and the BT group. Unlike the family, this genus is included in the regression model for blood pressure with (Table 2) and without ROO [17].

Finally, the genus *Desulfovibrio* is also noteworthy, not so much for its correlations but rather for the lack of them when ROO data are considered. This genus presents a statistically significant difference in percentage between the EVOO and BT diets and has a distinct profile in ROO too, although without achieving significance with respect to any of the other diets (Figure 5). The genus percentages in the different diets already have the profile that will be attained at the end of the experiment, after 12 weeks of diet [16], when EVOO and ROO are significantly different, with its coefficient being statistically significant in the multiple regression models for FI, WI, diuresis, and total cholesterol. At six weeks, some of these correlations (12-week FI and WI, together with 12-week SBP) are drawn when compared to butter and standard diets [17], but when ROO data are compared, they no longer apply. It could be possible that this circumstance is due to the presence of the genus *Rikenella*, with a very similar percentage in the four diets but about ten times more prominent, diminishing the significance of *Desulfovibrio* in the regression model for SBP, but other explanations can also be found and added as well. *Desulfovibrio* is a sulfate-reducing bacterium that uses sulfur-reduced species as the last electron acceptor instead of oxygen. Compounds as such can be found in a butter-enriched diet [17,23,24] but they are not expected in ROO. However, *Desulfovibrio desulfuricans* has also been reported to reduce nitrates and nitrites as alternative electron acceptors to sulfates to support growth [25], and nitro-oleic acid has recently been detected in olive oil [26]. This and other possible oxidized species would explain the relatively high percentage of *Desulfovibrio* in ROO at six weeks and also the worse relation to blood pressure when data from ROO is considered, both at six (this work) and 12 weeks [16], since these alternative electron acceptors will not produce H_2_S as a reduced outcome and will not participate in some of the mechanisms hypothesized for this bacterium to contribute to a blood pressure increment in the long run [17], although other negative effects, such as total cholesterol, FI, WI, and diuresis, still persist [16]. In any case, the different behavior of this genus in the EVOO and ROO diets confirms the role of the virgin olive oil polyphenols in inhibiting the growth of *Desulfovibrio* in the EVOO diet; this was even more evident over the full term of the experiment when the percentages of this genus in the BT and ROO diets had doubled and EVOO and ROO had significantly different values [16].

## 4. Materials and Methods

### 4.1. Animals

Experimental procedures were followed as already described [9,16,17]. Thirty-five (six-week-old) male Swiss Webster ICR (CD-1) mice (Harlan Laboratories) weighing 30.1 ± 0.55 g at the beginning of the study were fed ad libitum for 12 weeks a freely available standard diet (SD; standard laboratory mice diet A04, 3% fat, Panlab, Barcelona, Spain) (*n* = 8) or three high-fat diets (35% total energy) containing SD supplemented with 20% either butter (*n* = 9) (BT), extra virgin olive oil (*n* = 9) (EVOO) or refined olive oil (ROO), respectively (Table 3) [9,16].

EVOO was obtained from a fully organic crop (Soler Romero, Alcaudete, Spain). Butter and ROO were obtained from a large commercial store (Hacendado, Mercadona, Jaén, Spain). Fatty acid percentage and characterization were performed (EVOO 78.6% monounsaturated fatty acids -MUFA-, 4.2% polyunsaturated fatty acids -PUFA-, 17.1% saturated fatty acids -SFA-; butter 35.6% MUFA, 1.5% PUFA, 62.5% SFA; ROO 76.6% MUFA, 7.1% PUFA, 16.3% SFA; SD 0.5% MUFA, 1.75% PUFA, 0.75% SFA); and EVOO polyphenol content was obtained from the producer (total polyphenol content was 527 mg/kg). All experimental procedures were performed in accordance with the European Communities Council Directive 86/609/EEC and reviewed and approved by the Bioethics Committee of the University of Jaén, initially on 29 December 2010 for project AGR 6340 and extended for project PP2015/08/09. The procedure was followed as described previously [9,16]. Mice were housed at a constant temperature (23 °C), constant humidity (50%), and a constant day length (12 h). Animals were individually housed in metabolic cages twenty-four hours at six weeks of the experiment [17] and food intake, water intake, diuresis, body weight (BW), and systolic blood pressure (SBP) were measured individually. In addition, at that moment, feces were also collected individually right after deposition, and total DNA was extracted immediately as indicated in Section 4.2 or kept at −80 °C until use. SBP was monitored by the pleithysmographic method in unanesthetized animals as previously described [9,27]. Briefly, mice were placed in plastic holders and warmed to 37 °C for each recording session. At least seven determinations were made in every session, and the mean of the stable values within a range of 5 mmHg was recorded as the SBP level. Measurements at the beginning and end were discarded. Animals were kept in cages for six more weeks; the procedure was repeated at the end of the experimental period, and blood samples were obtained as described and already reported [9,16], obtaining the values of insulin, fasting glucose, triglycerides, total cholesterol, HDL, leptin, and ghrelin [28,29,30]. All analyses were performed according to the manufacturer’s protocols.

### 4.2. Bacterial Biodiversity

In order to study the bacterial composition in feces, DNA was extracted using the QIAamp^©^ DNA Stool Kit (QIAGEN, Hilden, Germany) as described and reported previously [9,16,17]. Thirty-five DNA samples, corresponding to eight fecal samples from eight mice under SD and twenty-seven fecal samples from nine mice fed high BT, nine fed a high EVOO diet, and nine fed a high ROO diet, were pyrosequenced at Lifesequencing (Valencia, Spain) using Roche GS-FLX-Titanium + 454 pyrosequencing technology, targeting the 16S ribosomal DNA V3–V4 region (V3fwd: 5′-TCCGTCAATTYMTTTRAGT-3′, V4rev: 5′-CCTACGGGAGGCAGCAG-3′). Thermal cycling consisted of initial denaturation at 94 °C for 2 min followed by 30 cycles of denaturation at 94 °C for 20 s, annealing at 50 °C for 30 s, and extension at 72 °C for 5 min. Thirty-five libraries were constructed, the different amplifications were individually measured, and the quantity of amplified DNA was estimated using Quant-iTTM PicoGreen (Invitrogen, Waltham, MA, USA). After the quality filter was applied and sequences were trimmed and checked for quimeras using the UCHIME v. 4.2.40 program, the resulting sequences were assigned to different taxonomic levels using the Ribosomal Database Project Classifier. Rarefaction curves were obtained for each sample, and taxonomical levels were analyzed in order to confirm that no more taxonomical groups were expected to be found if sequencing was increased.

### 4.3. Statistical Studies

For the statistical analysis, we followed the procedures described previously [9,16,17]. Whenever the ANOVA assumptions are not met to test the equality of the means according to the different types of diet, we used the Kruskal–Wallis test. Where significant differences were detected, the Dunn test for pairwise multiple comparisons with Bonferroni correction was used. The significance level considered in all tests was 5%. All computations were done using R 4.1.2 (Auckland, New Zealand). In addition, multiple regression models have been fitted, considering the physiological variables as dependent variables and those that have shown statistically significant differences according to the diets as explanatory variables (all regression models were fitted using the open-source statistical package Gretl 2019c, San Diego, CA, USA).

## 5. Conclusions

In summary, there are several bacterial taxa that are significant in regression models for physiological variables related to metabolic syndrome, both in a concomitant way and also after several weeks of high-fat diet feeding. Among these variables, blood pressure is remarkable because its values at twelve weeks can be explained by a multiple regression model with some of these bacteria, but no such model was found for SBP values at six weeks of diet. The different prevalence of some of these bacteria under the three high-fat diets can be easily explained just by the type of fat that is present in the diet, as is the case with the family *Sphingobacteraceae* and related taxa, and, therefore, their involvement in the development of the high values in the systolic blood pressure is doubtful. On the other hand, differences in the genus *Desulfovibrio* under the three HFDs can be better explained if a contribution of this bacterium to the increment of blood pressure values is assumed and the antimicrobial role of the virgin olive oil polyphenols is presumed in the maintenance of low levels of this genus under an EVOO high-fat diet.

## Figures and Tables

**Table 1:** Regression fits for each of the physiological variables studied using as independent variables those families that show statistical differences in the percentage of sequences retrieved from fecal samples at six weeks. BW, body weight; FI, food intake, WI, water intake; DIU, diuresis; SBP, systolic blood pressure; Glu, glucose.

	BW		FI		WI		DIU	SBP		Glu
	6 w	12 w	6 w	12 w	6 w	12 w	6 w *	6 w	12 w	12 w
	0.36 (0.0007)	0.20 (0.0333)	0.27 (0.0065)	0.49 (0.0013)	0.15 (0.0309)	0.38 (0.0143)	0.14 (0.0470)	0.56 (0.0012)	0.45 (0.0002)	0.34 (0.0165)
*Rikenellaceae*					0.33 ±0.14 (0.0309)	0.57 ±0.26 (0.0359)				−5.61 ±2.53 (0.0344)
*Lactobacillaceae*			1.16 ±0.36 (0.0032)	1.28 ±0.37 (0.0019)						
*Sphingobacteriaceae*		0.61 ± 0.27 (0.0304)				−1.52 ± 0.58 (0.0143)		8.53 ±3.16 (0.0134)	11.83 ±2.56 (0.0000)	
*Spiroplasmataceae*		3.72 ± 1.76 (0.0426)						39.50 ±13.03 (0.0064)		
*Gracillibacteraceae*	10.66 ±2.80 (0.0006)		−3.54 ±1.31 (0.0110)	−5.42 ±1.38 (0.0005)						
*Rhodospirillaceae*				7.48 ±2.66 (0.0094)		24.39 ±10.24 (0.0252)	2.01 ±0.96 (0.0469)			
*Christensenellaceae*				18.90 ±7.79 (0.0220)					345.67 ±147.60 (0.0265)	
*Acetobacteraceae*	39.77 ±15.86 (0.0174)			−37.33 ±11.97 (0.0042)		−136.62 ±43.73 (0.0045)				
*Staphylococcaceae*								68.92 ±23.83 (0.0087)		
*Streptococcaceae*								543.38 ±119.17 (0.0002)		891.41 ±235.38 (0.0007)

For each case, regression coefficient estimate, s.e. and *p*-values are shown. R^2^ and *p*-values of the model are also indicated under each physiological variable. * Indicates that logarithms of data have been used for the analysis.

**Table 2:** Regression fits for each of the physiological variables studied using as independent variables those genera that show statistical differences in the percentage of sequences retrieved from fecal samples. BW, body weight; FI, food intake, WI, water intake; SBP, systolic blood pressure.

	BW		FI		WI	SBP	Glucose	HDL/LDL	Leptin
	6 w	12 w	6 w	12 w	12 w *	12 w *	12 w	12 w *	12 w
	0.36 (0.0008)	0.20 (0.0327)	0.39 (0.0013)	0.31 (0.0097)	0.19 (0.017)	0.51 (0.0002)	0.15 (0.0246)	0.53 (0.0001)	0.37 (0.0003)
*Alistipes*			0.14 ±0.06 (0.0188)					0.22 ±0.05 (0.0004)	
*Lactobacillus*			1.26 ±0.34 (0.0008)	1.11 ±0.38 (0.0072)					
*Parapedobacter*		0.64 ±0.28 (0.0298)				0.06 ±0.02 (0.0011)			
*Spiroplasma*		3.56 1.74 (0.0497)				0.18 ±0.07 (0.0209)			
*Gracillibacter*	0.68 ±0.07 (0.0009)		−3.21 ±1.22 (0.0134)	−4.36 ±1.47 (0.0059)					
*Rikenella*						0.06 ±0.02 (0.0075)			
*Christensenella*				18.90 ±8.64 (0.0366)					
*Anaerophaga*									3827.14 ±918.48 (0.0003)
*Oleomonas*	1.01 ±0.39 (0.0151)				−6.83 ±2.69 (0.0171)				
*Staphylococcus*							145.68 ±61.76 (0.0246)		

For each case, regression coefficient estimate, s.e. and *p*-values are shown. R^2^ and *p*-values of the model are also indicated under each physiological variable. * Indicates that logarithms of data have been used for the analysis.

**Table 3:** Nutrient composition and energy content of standard (SD) and high-fat diets enriched with extra virgin olive oil (EVOO), refined olive oil (ROO), and butter (BT) [7,11].

Diet	SD		EVOO		ROO		BT	
	g/100 g	% Energy	g/100 g	% Energy	g/100 g	% Energy	g/100 g	% Energy
Protein	16.5	20	16.5	14	16.5	14	16.5	14
Carbohydrates	60	72	55	48	55	48	55	48
Fat	3	8	20	35	20	35	20	35
Total Energy (kJ/g)	14.2		19.6		19.6		19.6	

## Data Availability

Not applicable.
